# Preparations for Invasion: Modulation of Host Lung Immunity During Pulmonary *Aspergillosis* by Gliotoxin and Other Fungal Secondary Metabolites

**DOI:** 10.3389/fimmu.2018.02549

**Published:** 2018-11-06

**Authors:** Maykel Arias, Llipsy Santiago, Matxalen Vidal-García, Sergio Redrado, Pilar Lanuza, Laura Comas, M. Pilar Domingo, Antonio Rezusta, Eva M. Gálvez

**Affiliations:** ^1^Instituto de Carboquímica ICB-CSIC, Zaragoza, Spain; ^2^Immune Effector Cells Group, Aragón Health Research Institute (IIS Aragón), Biomedical Research Centre of Aragón (CIBA), Zaragoza, Spain; ^3^Department of Biochemistry and Molecular and Cell Biology, Fac. Ciencias, University of Zaragoza, Zaragoza, Spain; ^4^Servicio de Microbiología - Hospital Universitario Miguel Servet, Zaragoza, Spain; ^5^Department of Microbiology, Preventive Medicine and Public Health, University of Zaragoza, Zaragoza, Spain

**Keywords:** aspergillus, pulmonary aspergillosis, secondary metabolism, Host Lung Immunity, Gliotoxin

## Abstract

Pulmonary aspergillosis is a severe infectious disease caused by some members of the *Aspergillus* genus, that affects immunocompetent as well as immunocompromised patients. Among the different disease forms, Invasive Aspergillosis is the one causing the highest mortality, mainly, although not exclusively, affecting neutropenic patients. This genus is very well known by humans, since different sectors like pharmaceutical or food industry have taken advantage of the biological activity of some molecules synthetized by the fungus, known as secondary metabolites, including statins, antibiotics, fermentative compounds or colorants among others. However, during infection, in response to a hostile host environment, the fungal secondary metabolism is activated, producing different virulence factors to increase its survival chances. Some of these factors also contribute to fungal dissemination and invasion of adjacent and distant organs. Among the different secondary metabolites produced by *Aspergillus* spp. Gliotoxin (GT) is the best known and better characterized virulence factor. It is able to generate reactive oxygen species (ROS) due to the disulfide bridge present in its structure. It also presents immunosuppressive activity related with its ability to kill mammalian cells and/or inactivate critical immune signaling pathways like NFkB. In this comprehensive review, we will briefly give an overview of the lung immune response against *Aspergillus* as a preface to analyse the effect of different secondary metabolites on the host immune response, with a special attention to GT. We will discuss the results reported in the literature on the context of the animal models employed to analyse the role of GT as virulence factor, which is expected to greatly depend on the immune status of the host: why should you hide when nobody is seeking for you? Finally, GT immunosuppressive activity will be related with different human diseases predisposing to invasive aspergillosis in order to have a global view on the potential of GT to be used as a target to treat IA.

## General introduction

The genus *Aspergillus* comprise different saprophytic fungal species with a high environmental prevalence that, under specific circumstances, might infect humans and other animals causing different infectious diseases. Among them *Aspergillus fumigatus* is a well-known human pathogen, responsible for an important morbimortality in immunocompromised and immunocompetent patients like cancer, transplanted, COPD and critically ill patients (1–3). It causes several diseases including invasive aspergillosis (IA), chronic pulmonary aspergillosis (CPA) and allergic bronchopulmonary aspergillosis (ABPA) (4).

Among them IA is a common cause of mortality in patients with hematological malignancies and it is an emerging problem for solid organ transplant recipients, critical care patients and those receiving immunomodulatory therapies, with mortality rates ranging between 30 to 90% (1–3).

In order to colonize the host, *A. fumigatus* must use different evasion strategies to avoid the host protective response. These include physicochemical and anatomical barriers of the respiratory track like enzymes, mucus or epithelial cells as well as others that prevent spore and hyphae clearance by innate and adaptive immune system. Among these strategies the production of mycotoxins and other substances with immunosuppressive activity has been the focus of extensive research during the last years, although in most cases, the biological relevance of the findings has not been completely clarified. In this short review we will first summarize the main strategies used by the host to fight *Aspergillus* within the respiratory track, focusing on cellular innate and adaptive immune responses. Subsequently, we will present the main mycotoxins and products of the secondary metabolism with potential immunosuppressive activity. We will pay special attention to Gliotoxin (GT) that has been shown to affect a great variety of innate and adaptive immune responses and act as a virulence factor *in vivo* in mouse models (5). Finally, we will discuss unsolved questions and future directions to be addressed on the field, with special attention in the potential of immunosuppressive mycotoxins to exacerbate infection (act as virulence factors) depending on the immunosuppressive host status.

## Host lung immunity against aspergillus

The respiratory system is formed by the upper respiratory tract, nasal cavity, pharynx, larynx, the lower respiratory tract, trachea, bronchi, bronchioles and the respiratory zone represented by alveoli. To carry out gaseous exchange, the respiratory system is exposed daily to thousands liters of air, introducing numerous particles and potentially harmful microorganisms to the alveolar surface (6). To avoid injuries and infections, the respiratory tree has various defense mechanisms such as cough and the mucociliary transport system, formed by four major cell types that produce a physico-chemical barrier against microorganisms, including ciliated cells, mucus-secreting cells and basal cells (7). Nevertheless, if the potentially harmful microorganisms manage to overcome these elements, the bronchial tree still presents different defense mechanisms consisting of soluble molecules and humoral and cellular factors belonging to the innate and adaptive immune system.

Inhalation of *Aspergillus* spp. conidia is very frequent, because *Aspergillus* species are found in decomposing vegetation, soil, water, food and air. However, immunocompetent individuals are capable to eliminate *Aspergillus* conidia by different immune mechanisms, preventing germination and fungal growth (8, 9) (Figure).

### Innate immune response against *aspergillus*

Resident alveolar macrophages (AM) and epithelial cells interact with germinating *Aspergillus* spores in the lung. These cells recognize pathogen-associated molecular patterns (PAMPs) present in fungal surface like galactomannan and β-1,3-glucan among others, through pathogen-recognition receptors (PRR) such as Toll-like receptors (specially TLR-1,−3,−4, and-6), the C-type lectin receptor-Dectin-1 (9) or Nod-like receptors (10). *Aspergillus* recognition leads to the generation of proinflammatory cytokines like IL-1α, IL-1β, TNF-α, IL-8, and MIP-1α by activation of the NFkB and inflammasome pathways (10–12).

AM are also capable of eliminating directly conidia and initiate an inflammatory response to fungal infection. AM phagocytose conidia and kill them using different mechanisms including acidification of the phagolysosome and activation of antimicrobial enzymes (cathepsin D and chitinase), and the production of reactive oxygen species (ROS) (13, 14). Chemokines and proinflammatory cytokines act as chemoattractants and activators for other immune cells including neutrophils, Natural Killer and T cells that will arrive to the infected site to fight infection and prevent host colonization. The role of these receptors and cytokines in humans mainly proceed from studies showing a higher risk of IA in patients presenting Singe Nucleotide Polymorphisms (SNPs) for these genes (15, 16). Paradoxically, others like NOD2 SNP, decrease the risk of IA in Stem-Cell transplanted (SCT) patients (17).

After epithelial cells and resident AM initiate the inflammatory response, neutrophils are among the first cells arriving at the infected site. These cells have also been found to be critical during the immune defense against *Aspergillus* spp. both mouse models and humans. This role was mainly characterized in patients treated with neutropenia-inducing drugs as well as in those presenting mutations in molecules involved in neutrophil activity like NADP oxidases (18). Neutrophils are attracted to the site of infection by chemokines and cytokines, especially IL-8 and IL-17, albeit as indicated below, the role of IL-17 during IA is not clear (19–23). Apart from enhancing the inflammatory response by producing cytokines and chemokines, these cells can directly phagocyte and kill the fungus by the production of ROS and antimicrobial compounds (24). Neutrophils have another antifungal mechanism, the neutrophil extracellular traps (NET). NETs are formed when neutrophils release DNA, histones, and granular proteins, including calprotectin and PTX3 into the surrounding environment after autolysis, avoiding the progression of infection (24). Indeed, a recent study has shown that Stem Cell transplanted patients with a SNPs in PTX3 present a higher risk of IA (25). The role of NET formation in IA has also been demonstrated in patients with chronic granulomatous disease who received a gene therapy to restore NET and may resolve a preexisting pulmonary aspergillosis (26). Activated neutrophils also amplified immune response producing cytokines like IL-12 and IL-18 (24).

AM and neutrophils express cytokines and chemokines that attract antigen-presenting cells (APC) like dendritic cells (DC) and monocytes from the blood and surrounding tissues to the infection site. DCs link innate and adaptive immune response to fungal infection (27). They are a heterogeneous population characterized by the expression of different specific surface markers. Three main groups have been established: conventional DCs (cDCs), plasmacytoid DCs (pDCs), and monocyte-derived dendritic cells (moDCs). DCs are responsible for capturing, processing and presenting antigens associated to HLA-I/-II (MHC-I/-II) to CD8^+^ and CD4^+^ T cells, respectively, in the lymph nodes. This interaction leads to the generation of CD4^+^ Th-1, Th-2, Treg or Th17 subsets that regulate different immune responses. DCs also provide co-stimulatory signals (CD86, CD80) and secrete IL-12, a cytokine necessary for acquisition of cytotoxic activity in CD8 T^+^ cells (28). DCs have also been involved in NK cell activation during IA by the expression of SYK and IL2RA (29). It has been reported that pDCs play an important role *in vivo* during the control of infection, since its depletion in mice increased susceptibility to IA (30). Intriguingly, these authors also demonstrated that pDCs were able to directly inhibit the growth of *A. fumigatus* hyphae.

During *Aspergillus* infection monocytes migrate to the lungs where they differentiate into moDC. It has been demonstrated that moDCs are important for the maintenance and development of protective Th1 cell response against *A. fumigatus* (31). Monocytes are capable to recognize PAMPs in conidia and hyphae during *A. fumigatus* infection increasing the expression of several cytokines and chemokines. It has been recently described a new member of the C-type lectin receptor family, MelLec, expressed by endothelial, epithelial and myeloid cells, that recognizes DHN-melanin in *Aspergillus* conidia and is critical for host protection in a mouse model of IA (32). The relevance of these findings in humans was provided by showing that a SNP in MelLec increased the risk of IA in SCT patients.

In addition, it was found that monocytes may contribute to thrombosis and local lung tissue injury during *A. fumigatus* infection, increasing the expression of urokinase type plasminogen activator (uPA), urokinase type plasminogen activator receptor (uPAR), plasminogen activator inhibitor (PAI), pentraxin-3 (PTX3) and intercellular adhesion molecule-1 (ICAM-1) (33). Some evidence indicates that Natural Killer cells (NK cells) are involved in the control of *Aspergillus* infection. *In vitro* studies have demonstrated that NK cells exhibit antifungal activity against hyphal form of *A. fumigatus* but are not able to exhibit fungicidal activity against conidia (34). Another study reported that antifungal activity of NK cells against *Aspergillus* was IFN-γ-mediated and was independent of their cytotoxic mechanisms (35). *In vivo* studies have shown the important role of NK cells during *Aspergillus* infection. In a mouse model of Aspergillosis in neutropenic mice it has been demonstrated the beneficial effect of transference of NK cells (36). NK-cell-derived interferon (IFN)-γ also contributes to control infection activating macrophage-dependent fungal clearance mechanisms (37). NK cells have been shown to interact with neutrophils. NK cells activated by *Aspergillus* express TNF-α, IFN-γ and GM-CSF which directly stimulate neutrophil activation (38). However, so far it has not been identified any genetic deficiency, like Natural Cell Receptors SNPs, linking NK cells with IA susceptibility in humans, which would confirm such a role.

Other innate immune cells including mast cells, basophils, and eosinophils may contribute to fungal protection. The role of mast cells in *Aspergillus* infection is poorly understood. An *in vitro* study found that *A. fumigatus* hyphae induced degranulation of mast cells via an IgE-independent mechanism (39). However the biological relevance of this finding remains to be established (40). The role of eosinophils in *Aspergillus* infection has been established using mice that exhibit a selective deficiency in eosinophils. These mice showed impairment in *A. fumigatus* clearance and evidence of germinating organisms in the lung (41).

### Adaptive immune response against *aspergillus*

The innate immunity response during *Aspergillus* infection triggers the development of an acquired immune response inducing the differentiation of CD4 T helper cells into Th1, Th2, Th17, or Treg cell phenotypes which contribute to IA protection. However, the relative role of each subset is still a matter of controversy.

Th1 cells may improve the antifungal activity of macrophages and neutrophils in the site of infection throw the expression of proinflammatory cytokines TNF-α and IFN-γ (42). Furthermore, in healthy individuals it has been demonstrated the predominance of Th1 response against *A. fumigatus* employing peripheral blood (43). On the other hand, Th2 cells do not seem to play a protective role during *A. fumigatus* infection. In contrast, these cells may activate M2 macrophages and decreased Th1 cell response, which could be detrimental in patients with severe fungal infections (44). In contrast, in patients with allergic bronchopulmonary aspergillosis (ABPA), *A. fumigatus*-specific Th2 CD4^+^ T cells are predominant (45) and a recent work has identified SNPs in genes related with Th2 responses like *IL13* and *IL4R* that increase ABPA susceptibility (46).

The role of Th17 cell response during *A. fumigatus* infection is controversial. In a mouse model of *A. fumigatus* infection, it has been reported that IL-17 and IL-23 do not play a protective role due to its ability to negatively regulate the development of Th1 cells and to affect the neutrophil antifungal activity *in vitro* (19). Supporting this conclusion they showed that *in vivo* blocking of IL-23 and IL-17 increased infection clearance (19). In contrast, another mouse model of *A. fumigatus* infection showed a protective role of IL-17. In this model, *in vivo* neutralization of IL-17 early during infection increased fungal pulmonary burden (21). Concerning humans, it was found low frequency of IL17 producing cells and high frequency of IFN-γ producing cells after Ag-stimulation of PBMCs from healthy donors (22) or from patients with IA (23). Intriguingly, the last work also found a marked induction of IL-10 producing cells, which could modulate the generation of specific CD8^+^ T cell activity among other responses. More recently, it was found that meanwhile T cells from peripheral blood from IA patients showed a Th1 IFN-γ producing profile, the majority of lung-derived *Aspergillus*-specific T-cells displayed a Th17 phenotype, and only low percentages of cells produced IFN-γ. However, it has been shown that SNPs in the IFN-γ gene increases the susceptibility to IA in SCT patients (16). These results indicate that during *A. fumigatus* infection both Th1 and Th17 cell responses may play an important role in host immunity.

Treg cells may play a protective role during *A. fumigatus* infections modulating the exacerbated inflammation due to a strong Th1 response in early stage of *A. fumigatus* infection as well as hypersensitivity reactions associated with Th2 responses in later stages (47, 48).

CD8^+^ T cell response may play a protective role during *A. fumigatus* infection. In a mouse model of *A. fumigatus* infection, it has been observed an increment of IFN-γ-producing CD8^+^ T cells in bronchoalveolar fluids of mice repeatedly challenged with *A. fumigatus* conidia with the maintenance of airway memory phenotype CD8^+^ T cells (49). However, functional evidences of such role were not investigated.

## Immunosuppressive activities of aspergillus secondary metabolism

*Aspergillus* species produce a large number of secondary metabolites that are not critical for its life cycle, but confer competitive survival advantages. These metabolites include aflatoxins, naptho-γ-pyrones, ochratoxins, cyclopiazonic acid, fumonisins, patulin, gliotoxin, kojic acid, malformins, emodin, bicoumarins, csypyrone B1, DHBA, nitropropionic acid, aflatrem, ophiobolins, etc. Many of these compounds exhibit interesting biological properties like antibiotic, anti-carcinogenic or anti-inflammatory activity (50). Thus, *Aspergillus* spp. are used as biological factories with a great range of applications in food, textile or pharmaceutical industry.

Within these metabolites, mycotoxins have focused special attention due to its toxicity, carcinogenic and/or immunosuppressive activity for both humans and livestock. Among them, fumifungin, fumiquinazoline A/B and D, fumitremorgin B, gliotoxin, sphingofungins, pseurotins, and verruculogen are found in *A. fumigatus*, being gliotoxin (GT) the most abundant and best characterized mycotoxin produced by *A. fumigatus* (51).

In Table 1 the main secondary metabolites and mycotoxins with immunosuppressive activity are summarized, including the main *Aspegillus* spp. producing them and the effect on host immunity. Notably, aflatoxins, ochratoxin, and gliotoxin are the most extensive studied and, thus, for which more immunosuppressive activities have been described. It is worth to note that in all cases these compounds mainly affect innate macrophage and neutrophil responses, especially the pro-inflammatory response, highlighting the importance of these cells in the elimination and prevention of *Aspergillus* infection as described above.

**Table 1:** Main secondary metabolites from *Aspergillus* spp. presenting immunosuppressive activity.

**Metabolite**	***Aspergillus* spp**.	**Affected immune function**	**References**
Aflatoxins	*A. flavus, A. parasiticus, A. niger*	Macrophage Neutrophil Cytokine production T cell number	(52–57)
Ochratoxin A	*A. niger, A. ochraceus, A. carbonarius, A. alliaceus, A. sclerotiorum, A. sulphureus, A. albertensis, A. auricomus, A. wentii*	Immune organ reduction Ab production Cytokine production T cell death	(52)
Fumagillin	*A. fumigatus*	Neutrophils	(58)
Fumonisins	*A. niger*	Dendritic cells(Ag presentation Th1/Th2 balance Macrophage/ Cytokne production	(52)
Patulin	*A. clavatus*	Macrophage/ Cytokne production T cell/ Cytokne production	(59–61)
Citrinin	*A. carneus, A. terreus*	Macrophage / cytokine production T cell/ Cytokne production	(53, 60, 62, 63)
Malformins	*A. niger*	IL1b activity	(48)
Emodin	*A. ochraceus, A. wentii*	Macrophage/Cytokine production	(64, 65)
Sterigmatocystin	*A. nidulans, A. versicolor, A. flavus*	T regulatory cell increase Dendritic cell reduction Neutrophil/NADPH oxidase	(66, 67)
Cytochalasins	*A. clavatus*	Macrophage phagocytosis Neutrophil/phagocytosis/chemotaxis T cell/activation NK cell/activation	(68–70)
Gliotoxin	*A. fumigatus, A. flavus*	Macrophage and monocytes / phagocytosis, cytokine production Neutrophil / NADPH oxidase, phagocytosis, migration Eosinophil / apoptosis Mast cell / degranulation, cytokine production Dendritic cell / Maturation, Ag presentation T cell / activation NK cell Immune cell / apoptosis	(71–73)

In several cases the immunosuppressive activity of these compounds has been related to its toxicity against immune cells like some aflatoxins, ochratoxin, gliotoxin or sterigmatocystin. Ochratoxin A is toxic for several immune cells *in vitro* as well as *in vivo* in different animal models, causing the reduction in the size of different immune organs including spleen, tonsil or lymph nodes (52). Sterigmatocystin has also been described to be toxic for dendritic cells causing a reduction in its number *in vivo* (66, 67).

More interestingly, other mycotoxins can affect different immune responses at a concentration that do not cause cell toxicity. For example, citrinin and aflatoxin B1 inhibit NO production in macrophages without cell death (53). As indicated, a common feature of most of these compounds is its ability to inhibit inflammatory cytokine production by macrophages by blocking different mechanisms like TLR expression, RIG or NFkB activation, all of which are involved in the synthesis of cytokines following a pro-inflammatory stimulus (Table 1). Thus, some of them like gliotoxin or emodin have been proposed as anti-inflammatory agents to treat different pathologies like septic shock or colitis in mouse models (64, 65, 74). However, its application for humans is still pending and might be very difficult due to likely secondary toxic effects, unless they are formulated in compositions that allow local selective delivery in affected tissues.

In addition to macrophages, patulin has been shown to directly affect T cell responses, affecting the polarization between Th1 and Th2 by a mechanism dependent on intracellular glutathione (59). Fumonisin prevents dendritic cell maturation and antigen presentation, blocking antigen-specific T cell responses (52). Thus, fumonisin exposure might cause specific T cell immunosuppression and enhance susceptibility to intracellular pathogen infections like viruses, although this hypothesis has not been tested yet.

Finally, some of them might regulate very specific processes like malformin, that has been shown to inhibit IL-1β activity by preventing IL-1β binding to its receptor (75). Indeed, Malformin is commercialized as a specific IL-1β inhibitor. Since inflammasome activation and IL-1β production are critical for initiation of innate and adaptive immune responses, this compound could regulate a broad range of immune responses, including intracellular and extracellular pathogens. Concerning, inflammasome activity and IL1 production Emodin has been shown to inhibit inflammasome activation mediated by ATP and to prevent LPS-mediated septic shock in animal models (64).

## Immunosuppressive activity of gliotoxin

Despite the immunosuppressive properties described for several mycotoxins, as shown in Table 1, GT is the one that affects a wider variety of immune responses. This is likely because it has been the most extensively studied and characterized, since it is the most abundant mycotoxin produced by *A. fumigatus*, the main *Aspergillus* spp. causing IA.

One the main structural features of GT that regulates its biological activity, including toxicity (cell death) and immunosuppression, is the presence of a disulphide bond, conserved in most members of the epipolythiodioxopiperazine (ETP) family (76). GT can bind and inactivate proteins through cysteine residues and generate ROS thanks to the disulfide bridge present in its structure. It is believed that the generation of ROS and activation of the mitochondrial pathway by Bak is responsible for the toxicity of the GT (77) and is produced by redox reactions between the oxidized (GT) and reduced form (SH2-GT) (78). Morever, the entry of GT in tumor (71) and immune cells (79) has also been shown to be dependent on the presence of the intact disulphide bond.

**Figure 1:**
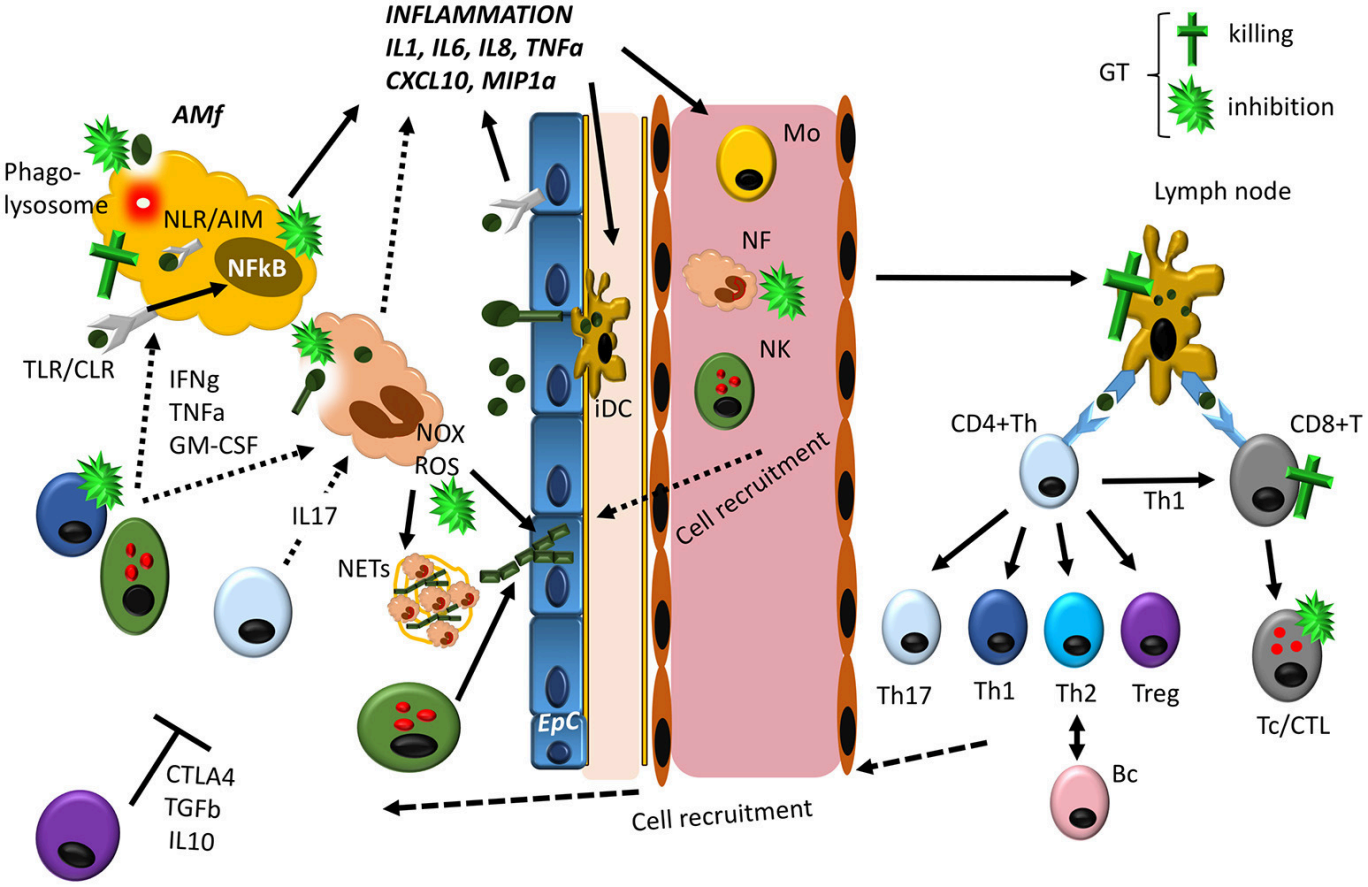
Overview of the lung immune response against Aspergillus, indicating the main targets for GT-induced immunosuppression. Resident alveolar macrophages (AM) and epithelial cells (EpC) interact with germinating *Aspergillus* spores in the lung by different PRRs (mainly TLRs, C-type lectin receptors/CLRs and NLRs), activating the NFkB transcription factor mainly responsible of the synthesis of inflammatory cytokines and chemokines. In addition, AM are able to directly kill phagocytosed spores in phagolysosomes. In response to inflammation, several cells are attracted to the infected site and activated like neutrophils (NF), monocytes (Mo) and NK cells. NF are the first cells extravasating from circulation to the infection site, where they phagocytose and kill Aspergillus conidia and enhance the inflammatory response. In addition, they are able to kill hyphae generated from conidia that have avoided AM, by releasing ROS produced by NADPH oxidase (Nox) as well as to trap them in structures released when neutrophils die known as NETs. Other cells like circulating Mo and NK cells also contribute to *Aspergillus* clearance, directly or by releasing cytokines that enhance anti-fungal activity of AM and NF. Meanwhile the innate immune response tries to eliminate *Aspergillus* conidia and hyphae, interstitial immature Dendritic Cells (iDC) phagocytose conidia and hyphae and migrate to lymph nodes, where fully mature DCs present these antigens to CD4+ Th and CD8^+^ T cells. Here depending on the nature of the Ag presented and the cytokines produced by DC, Th cells differentiate into the different subsets, CD8^+^ T cells are activated generating cytotoxic T cells (Tc/CTL) and B cells transformed in antibody producing plasma cells. All these cells migrate to the site of infection and contribute to the elimination of the fungus (Th1 and Th17 cells) and to avoid an exacerbated inflammatory response (Treg cells), by expressing cytokines and ligands with different activities. As described in the text GT can interfere with host immune response at different levels. The most pronounced effect seems to be related with its ability to block the inflammatory immune response of macrophages by direct killing or by inhibiting NFkB as well as phagocytosis. In addition, GT is able to kill epithelial cells, and, thus, the fungus could potentially use GT to completely inhibit the generation of the immune response. However, it should be noted that GT is only produced at the hyphae level, and, thus, AM and EpC will be able to activate the immune response, before hyphae are produced. At this stage NFs are critical to eliminate hyphae and, GT has been described as a potent inhibitor of NOX as well as phagocytosis. Finally, GT would inhibit the adaptive immune response at different levels, contributing to host colonization. From this scheme, it seems clear that the contribution of GT to *Aspergillus* infection will depend on the balance between host immune activity and hyphae development.

Most of the studies presented below concerning GT activity on specific cellular functions have been shown *in vitro*, although it is well known that GT also exerts immunosuppressive effects *in vivo* in mouse (80) and rat models (81). Indeed, it was suggested as a potential immunosuppressive drug during organ and bone marrow transplantation (82, 83). The mechanism involved seems to be related, at least in the mouse model, with the ability of GT to kill immune cells in spleen, thymus and lymph nodes. However, depicting the effects of GT *in vivo* against specific cell responses is challenging, and in most cases, it seems that they will be a consequence of its pleitropism to affect most of the immune responses involved in *Aspergillus* immunity (Figure 1).

### Monocytes and macrophages

In late '80s the group of Arno Mullbacher in Canberra observed in a culture of macrophages accidentally contaminated with a mold that cells spontaneously detached from plates and apparently, remained alive. Macrophages are known to be very difficult to detach from plastic surfaces without killing them, and, thus, the group decided to characterize the compound responsible for this activity (84). They identified it as gliotoxin. Subsequently they found out that it presented a variety of immunosuppressive activity *in vitro* in macrophages by preventing H_2_O_2_ production and bactericidal activity (85). And in antigen presenting cells (APCs) including anti-phagocytic and immunomodulating activity, preventing phagocytosis and activation of T cell responses, including cytotoxic CD8^+^ T cells (84). Notably, GT did not prevent T cell mediated cytotoxicity once cells were activated, confirming that GT modulated APC activation, preventing APC-mediated T cell activation.

Later on it was shown that apart from its immunomodulatory activity, GT was able to induce apoptosis in macrophages (86) by a mechanism involving ROS production, caspase activation and the intrinsic mitochondrial pathway in mouse macrophages and human monocytes (87). GT induces apoptosis in cultured macrophages via production of ROS and cytochrome c release without mitochondrial depolarization (88). Notably, this effect was not observed in neutrophils, although it does affect its phagocytic capacity (89). The ability of GT to kill macrophages has been related to the inhibition of macrophage function including phagocytosis and pro-inflammatory cytokine production in response to *Listeria monocytogens* infection (90) or LPS stimulation (62). Concerning the physiological relevance of these *in vitro* findings, it was shown that the ability of GT to inhibit macrophage function *in vitro* correlated with an increase replication of *Listeria in vivo* (90).

It should be noted here that the biological effects of GT on macrophage function, as well as against other immune cells, could be dependent on the concentration. At high concentrations most effects are related to the ability of GT to kill immune cells, meanwhile at lower concentrations specific immunosuppressive effects non-related to cell death could be observed (85).

Concerning the effects non-related to cell death, GT is a well-known inhibitor of NFkB activation in different cells including macrophages, which blocks the production of pro-inflammatory cytokines in response to different stimuli (81). However, it should be indicated that it is not a trivial question to find out a concentration that inhibits NFkB activation and pro-inflammatory cytokine production in macrophages, independently of GT-mediated killing, at least in mouse macrophages (Unpublished data), which might affect the proper interpretation of these findings. However, the relevance of these findings in humans is not clear, since a recent study has shown that SNP in different molecules of NFkB pathway do not increase the risk of IA in SCT patients (91). Thus, in order to clarify the role of GT-mediated NFkB inhibition during IA, studies comparing NFkB activity during infection with GT producing and non-producing *A. fumigatus* strains should be carried out.

If confirmed in relevant *in vivo* models, this finding could be a key in order to confirm GT as a prominent immunosuppressive virulence factor: blocking NFkB would affect host immunity early during infection, since this transcription factor is critical for the generation of the inflammatory response after activation of most PRRs involved in *Aspergillus* immunity including TLR and CLRs.

Another mechanism by which it has been recently described that GT affects macrophage phagocytosis is the interference with IP3 metabolism, which affects integrin activation as well as actin cytoskeleton remodeling, both of which are required for efficient phagocytosis (92).

### Neutrophils and other polymorphonuclear cells

Another key feature of GT, regarding its immunosuppressive activity, is the ability to affect several neutrophil functions in the absence of cell death. Here it should be noted that it was described that GT was not cytotoxic for human neutrophils at concentrations where monocyte/macrophages were readily killed (89). Thus, it seems that in this cell type the effects observed for GT can be clearly analyzed in the absence of cell death contribution and, as discussed below neutrophil inactivation might be the most relevant immunosuppressive function of GT during IA.

The first report on the immunosuppressive effect of GT on human neutrophil function was published when it was found that H_2_O_2_ production was reduced after GT exposure (85). Subsequently, a more detailed analysis of GT on neutrophil function revealed that it affected ROS production, but, in addition, inhibited phagocytosis. Notably, other functions like degranulation or myeloperoxidase activity were not affected (89). Inhibition of phagocytosis was confirmed by another independent study (93). The molecular mechanism behind the anti-oxidant activity of GT was solved in 2004, showing that GT disrupted the formation of a functional NADPH oxidase complex (94), a key finding concerning the ability of GT to interfere neutrophil function, since NADPH oxidase is critical for host protection against *Aspergillus*.

Intriguingly, it was shown that the effect of GT on neutrophils could be completely different in the presence of corticosteroids (89). In this case, GT increased ROS production in neutrophils treated with methyl-prednisolone, commonly used in patients at risk of IA, which could enhance inflammatory and tissue damage in non-neutropenic patients, a process that has been related with a high infiltration of neutrophils in lungs from *Aspergillus* infected patients. Thus, the contribution of GT during IA could be related not only to its ability to favor immune evasion, but, in addition, to an exacerbation of tissue damage induced by neutrophils in corticosteroid-treated patients.

Concerning other PMN cells, it was reported that inhibition of NFkB by GT increases eosinophil apoptosis mediated by TNF-α (95). However, the role of this inhibition during the interaction between host eosinophils and *Aspergillus* is unclear. On the one hand elimination of eosinophils could favor *Aspergillus* infection since these cells contribute to host defense against *Aspergillus*. In contrast, it has been recently reported that death eosinophils release NETs after interacting with *A. fumigatus* (96), which might contribute to *Aspergillus* clearance, although this hypothesis was not tested and remains to be solved.

NET formation is used by death neutrophils to trap microorganisms, facilitating its clearance and favoring the presentation of associated antigens by dendritic cells and the generation of adaptive immune responses. Recently, it was shown in a mouse model of pulmonary aspergillosis, that neutrophil NADPH oxidase activity is critical for NETosis and apoptosis during aspergillosis (97). Here it is tempting to speculate that GT could interfere with NETosis and *Aspergillus* clearance by inhibiting neutrophil NADPH oxidase, and thus, affect the transition from innate to adaptive immune system by reducing the amount of Ags available for DC uptake and processing. However, before all these hypotheses are experimentally addressed, the role of NETosis in *Aspergillus* killing should be clarified since a recent study indicates that NETosis is not a mechanism employed by human neutrophils to kill *Aspergillus* hyphae (98).

### Dendritic cells, antigen presentation, and T cell response

As indicated above, most immunosuppressive effects of GT have been related to innate immune responses, specially macrophages and neutrophils, in concordance with the key role of these cells during *Aspergillus* infection. However, adaptive immune responses, like T cells, have also found to be important for *Aspergillus* host defense by enhancing the activity of PMNs and macrophages (CD4 Th1 responses). The relevance of T cells in *Aspergillus* immunity has been shown in mice (99, 100) and human (101, 102, 103). Indeed, some patients undergoing specific therapies affecting T cell function also show increased susceptibility to *Aspergillus* infection, as in the case of solid organ transplantation (104).

Ag presentation and DC function have been shown to be modulated by GT by several independent groups, affecting subsequent T cell responses. Again, as in the case of macrophages, most effects seem to be related to the ability of GT to induce cell death on DCs. GT was found to kill monocyte-derived dendritic cells blocking Ag presentation and T cell activation, suppressing CMV specific T cell responses (87). In agreement with these findings it was also found that GT killed bone marrow derived DC inhibiting IL12 production and the generation of Listeria-specific CD8^+^ T cells (78). It was also found *in vivo* that GT eliminated Langerhans cells (LC), a type of skin associated DCs (105).

Apart from the ability to block T cell generation by affecting DC function, GT is able to directly kill and/or inhibit different T cell functions. Indeed, GT was shown to block NFkB activation in B and T cells by preventing IkBα degradation (106) and later on to kill CD8^+^ T cells, preventing cytotoxic T cell-mediated cytotoxicity (90). In contrast, at non-toxic doses, GT did not prevent CD8^+^ T cell function (107). Regarding cytotoxic T cell function, it was reported that GT inhibited CTL-mediated cell death by blocking granule exocytosis- and FasL-mediated cell death (108). The mechanism proposed for this action was the interference with CTL:target cell conjugation. Although authors argued that this defect was not due to GT toxicity on CTLs, from the results presented in that work it is not clear whether GT induced cell death on CTL or not. Notably this work contrast with previous findings indicating that GT did not prevent T cell mediated cytotoxicity once cells were activated (84).

In addition, GT has been shown to affect IFN-γ production by CD4^+^ T cells (60) which might reduce the ability of CD4^+^ T cells to enhance macrophage and neutrophil activity against *Aspergillus*.

### Natural killer cells and mast cells

Other cells from the innate immune system in which GT might have immunosuppressive activity are Natural Killer cells and Mast cells, both of which have been suggested to be involved in the control of *Aspergillus* infection (109).

However, meanwhile the evidences for a role of mast cells in *Aspergillus* immunity are mostly based on *in vitro* findings, NK cells have been shown to contribute *in vitro* (110) as well as *in vivo* (111, 112). However, up to date GT has not been shown to affect NK cell activity.

Concerning Mast cells, GT was shown to block both FcE receptor-dependent and independent activation including degranulation and lipid and cytokine production (113). The mechanism involved was related to the ability of GT to produce intracellular ROS in the absence of cell death.

### Unsolved questions and future perspectives

Although some secondary metabolites, especially GT, can contribute to infection and fungal colonization, as previously indicated (Table 1) most studies have been performed employing human and mouse *in vitro* cell models, and few *in vivo* evidences indicate a role for these metabolites in immune evasion and host colonization. An exception is GT, which was shown to act as a virulence factor *in vivo* in mouse models by employing *A. fumigatus* mutant strains genetically modified to delete specific genes involved in GT synthesis, like GliP or GliZ (5, 114–117).

However, a question that remains to be solved in humans, albeit it has been addressed in mouse models, is the fact that the role of GT as a virulence factor might be related to the immune status of the host; specifically, the absence of host immune cells that are targeted by GT in immunocompromised patients. In the studies mentioned above the results indicated that in mice treated with cyclophosphamide and corticosteroids, a combination that induces neutropenia, GT was unimportant for fungal virulence (114, 116). In contrast, in mice treated with corticosteroids, which just inhibit neutrophil activity without inducing neutropenia, GT synthesis significantly contributed to virulence (5, 117). Although this explanation has been accepted to reconcile the apparent contradictory results obtained in different studies, it is not completely clear whether this is the only difference to explain the contribution of GT to *A. fumigatus* virulence. Indeed, in mice treated with vinblastine, a chemotherapy drug that induces neutropenia, the mutant *A. fumigatus* GliP strain that did not produce GT, was less virulent than a wild type strain. However it should be indicated that neutrophil levels were not determined in these mice, albeit treatment was enough to promote infection (Pardo and Galvez, unpublished data).

Thus, it will be required further studies to solve whether GT only contributes to virulence in corticosteroid treated non-neutropenic host or whether it can also worsen IA evolution by affecting other immune cells involved in host defense such as macrophages, NK or T cells. Here it will be very interesting to test whether GT enhances virulence by promoting immune evasion and/or by enhancing neutrophil-mediated inflammatory tissue damage in corticosteroid-treated host as suggested (89).

In addition, GT could promote fungal invasion by affecting epithelial and/or endothelial cell barriers. Indeed, GT has been shown to kill lung epithelial cells *in vitro* (118). However, this hypothesis will require further experimental evaluation in mouse *in vivo* models.

Concerning humans, it will be very difficult to confirm whether GT actually contributes to virulence. Several groups have reported that most *A. fumigatus* strains isolated from humans are able to synthetize GT as well as the inactive derivative bmGT (79, 119) suggesting that at least *ex vivo* all fungal isolates synthetize GT, irrespectively of the host immune status from whom they were isolated (neutropenic or not). Confirming these *in vitro* findings, bmGT, which is synthetized from GT, has been identified in neutropenic (79, 120) and non-neutropenic (121) patients *in vivo*, suggesting that the fungus produces GT *in vivo*, even in situations where *a priori* should not be required (i.e., neutropenia). Here it should be noted that GT cannot be detected *in vivo* due to its high reactivity, and thus bmGT might be considered as a marker of GT synthesis. In order to confirm whether GT might help *Aspergillus* to colonize and invade human host, it will be required to analyze whether bmGT presence correlates with prognosis and survival in neutropenic and non-neutropenic patients.

Alternatively, even in situations where GT would not enhance *Aspergillus* virulence, it could promote, enhance and/or re-activate other infections by blocking macrophage, dendritic cell NK cell and/or T cell function like CMV, EBV or tuberculosis. Studies correlating GT (or bmGT) presence *in vivo* and risk of viral and/or bacterial co-infections will be required to solve this question.

## Author contributions

LS, SR, PML, LC, MPD, MV-G, and AR contributed to drafting the article. MA and EMG contributed to revising it critically and wrote the final version.

### Conflict of interest statement

The authors declare that the research was conducted in the absence of any commercial or financial relationships that could be construed as a potential conflict of interest. The handling Editor declared a shared affiliation, though no other collaboration, with several of the authors LS, PL, and AR.
